# Household structure is independently associated with malaria risk in rural Sussundenga, Mozambique

**DOI:** 10.3389/fepid.2023.1137040

**Published:** 2023-08-16

**Authors:** Kelly M. Searle, Dominique Earland, Albino Francisco, Valy Muhiro, Anisío Novela, João Ferrão

**Affiliations:** ^1^ Division of Epidemiology and Community Health, University of Minnesota School of Public Health, Minneapolis, MN, United States; ^2^ Escola Secondária de Sussundenga, Sussundenga, Mozambique; ^3^ Sussundenge-Sede Centro de Saude Rural, Sussundenga, Mozambique; ^4^ UniSCED Aberta de Mozambique, Chimoio, Mozambique

**Keywords:** malaria, housing, community health, epidemiology, Mozambique

## Abstract

**Introduction:**

Mozambique has the fourth highest malaria cases and malaria mortality globally. Locally, malaria incidence increases from low in the southern region to high in the central and northern regions. Manica Province in central Mozambique has the fourth highest prevalence of malaria out of the 11 provinces, and the highest in the central region of the country. In this area where coverage of interventions has been limited, household level risk factors can be important for understanding the natural history of infection, as well as the implementation of current and future interventions. There has been indication that the relationship between household structure and malaria risk is actually a mediating one between the true relationship between household income and education and *Plasmodium falciparum* infection. The objective of this study was to determine and quantify these complex relationships.

**Methods:**

We conducted a community-based cross-sectional study in Sussundenga village. Sussundenga is a rural village, located in Sussundenga District, Manica Province, Mozambique. We enrolled 303 participants from 83 randomly selected households. We collected information on demographics, household construction, and administered a *P. falciparum* rapid diagnostic test (RDT). We constructed several generalized estimating equations logistic regression models to determine the independent effects of housing construction on malaria risk. We also constructed models separate from generalized estimating equations logistic mediation models to determine the proportion of effects mediated by household construction material in the relationship between head of household occupation and education and malaria risk.

**Results:**

The overall malaria prevalence among the study population by RDT was 30.8%. In the multivariable model adjusting for all individual and household factors as potential confounders, rudimentary roof structure was the only household structural variable that was statistically significantly associated with increased malaria risk [OR 2.41 (1.03–5.63)]. We found no evidence that household structure mediated the relationship between head of household education or employment and malaria risk in our study population.

**Discussion:**

Household structure was a significant risk factor for malaria infection in our study population. These findings are consistent with malaria being a disease of poverty and an area that could be targeted for future interventions that could have long-term impacts.

## Introduction

Malaria represents an important global health problem and is concentrated primarily in sub-Saharan Africa (1). *Plasmodium falciparum* parasites are the causative pathogen that is associated with the most severe form of disease, which is the most prevalent species in this area. While malaria leads to high levels of morbidity and mortality it also perpetuates the cycles of poverty in impacted communities (2). It is well documented that individual factors, primarily age, are associated with risk for *P. falciparum* infection and subsequent disease (1). With increased age the risk for infection, morbidity, and mortality decrease with increased immunity (3, 4).

Over the past several decades there has been increased awareness and attention toward malaria control, elimination, and eradication (1). This increased awareness has been accompanied by increased funding for these efforts. The current interventions available to achieve such goals include, but are not limited to: insecticide treated bednet (ITN) distributions; indoor residual spraying (IRS) campaigns; case management through parasitological diagnosis and appropriate treatment with artemisinin combination therapy (ACT); and integrated community case management (iCCM) with community health workers (CHWs) conducting case management in areas with limited access to health facilities (1). Many countries that have increased coverage with these interventions have experienced marked declines in malaria incidence and prevalence as a result (5, 6). However there have been heterogeneities within and between countries in their responses to these interventions (6, 7). One country with heterogeneous transmission and varied distribution and responses to current interventions is Mozambique (8–10).

Mozambique has the fourth highest malaria cases and malaria mortality globally (1). Malaria incidence increases from low in the southern region to high in the central and northern regions (8, 9). Elimination efforts are underway in the south which borders the Republic of South Africa and Eswatini, which are also implementing elimination strategies (8, 9). The central and northern regions have had limited success with the current malaria control efforts (9, 11). Manica Province in central Mozambique has the fourth highest prevalence of malaria out of the 11 provinces, and the highest in the central region of the country (8, 9, 12). At the time of this study the malaria interventions in Manica Province were limited to ITNs and intermittent preventative treatment during pregnancy (iPTP), both distributed through antenatal care facilities.

In this area where coverage of interventions has been limited, household level risk factors can be important for understanding the natural history of infection, as well as the implementation and improvement of current and future interventions. Household structure, referring to the materials that are used for the roof, walls, floor, as well as windows and eaves, has been shown to be an incredibly important malaria risk factor (2, 13–17). This overall association between modern household structure and reduced malaria risk has held in several observational studies and led to community trials (18). The results of community trials however have been varied (18). This is likely because household structure is not readily defined, and condition of the structure is not commonly measured. A combination of modern and rudimentary roof, walls, floors, windows, and eaves structures make up most households in malaria endemic areas. Mixed associations have been found in different areas between different parts of the house and associations with malaria risk. Roof and floor structure are most associated, while windows and eaves have the most interventions planned upon as they are most convenient and least costly (2, 13, 16, 19). In reality each structure may have a different relationship with malaria risk depending on the environment and interventions should be planned accordingly.

There has been indication that the relationship between household structure and malaria risk is actually a mediating one between the true relationship between household income and education and *P. falciparum* infection (20). The underlying hypothesis in this relationship is that the head of household education level and income level are associated with household structure, which is then associated with *P. falciparum* infection. Partial mediation is also a hypothesis as education level of the head of household is also associated with risk of *P. falciparum* infection risk. From a research perspective, it is important to distinguish the difference between income and/or occupation and education as confounding factors in the relationship between household structure and *P. falciparum* infection, or as household structure as a mediating factor between the relationship between income and/or occupation and education and *P. falciparum* infection. The research findings can better inform how to apply interventions, and whether to focus on the more distal relationships, or more proximal, to be most effective. From a public health perspective, it is important to identify the modifiable factor most capable of preventing infection and having the largest impact in the long-term, whether that be income, occupation, education, or specifics of household structure. The objective of this study was to determine and quantify these complex relationships.

## Materials and methods

### Study area

We conducted our study in Sussundenga village. Sussundenga is a rural village, located in Sussundenga District, Manica Province, Mozambique. The village is approximately 70 km from the provincial capital of Chimoio, and 40 km from the Zimbabwean border. The climate is tropical with an average annual precipitation of 1,200 mm. There is a distinct rainy season that lasts from November—April, with a dry season from May—October (21, 22). The village is divided administratively in 17 residential areas called “bairros” (21). This area has perennial malaria transmission, with seasonal increases in *P. falciparum* malaria incidence during and following the rainy season. Sussundenga village is the district capital where the central municipal buildings and district level rural health center (RHC) reside. There is a central village for local commerce with primary and secondary schools. The local population is primarily agrarian with a population of approximately 20,000 inhabitants (21, 22).

### Data collection

Google Earth Pro™ satellite imagery was used to digitize and enumerate all household structures in the village of Sussundenga. A simple random sample of 125 households was taken with the goal of having 100 households for enrollment in the study and 25 households as backup for refusals and errors in the digitizing process (such as misclassified non-household structures). This was a pilot study, and the sample size was calculated based on the assumption of 5–6 residents per household and to distinguish between individual risk factors for *P. falciparum* malaria infection among residents. Coordinates of the households were extracted onsite using tablet computers and maps of the selected households to conduct study visits. Ethical review and approval for this study was completed by the Institutional Review Board (IRB) at the University of Minnesota [STUDY00007184] and from A Comissão Nacional de Bioética em Saúde (CNBS) at the Ministry of Health of Mozambique [IRB00002657]. This was a cross-sectional study that involved two visits to the selected households. The first was a notification visit where the study team introduced themselves to the head of the household and explained the objectives and procedures of the study. It is customary for the head of household to provide permission to the study team before any activities take place at the household involving other household members. Once the head of household gave permission, the study team conducted a household census with the head of household and began the process of individual informed consent with the household residents, for all adult (18+ years) residents and parental permission and assent from minors. After obtaining consent from the household residents, the study team informed participants when they would return the following day to conduct the study activities. The only eligibility requirement was that the residents live in household full time. Data collectors administered a questionnaire to collect individual demographics (e.g., age, sex, education, and occupation), recent malaria symptoms, use of an ITN the previous night, and healthcare access and use. The data collectors also administered a questionnaire to collect household level variables regarding the house construction materials, ownership of animals, and education and employment status of the head of household.

A study nurse collected current malaria specific symptoms by self-report. They then collected a finger prick blood sample to administer a rapid diagnostic test (RDT) (RightSign Biotest®, China). The results were recorded and in the event that a participant was positive for malaria the study nurse referred them to the Sussundenga RHC for diagnosis confirmation and treatment. The questionnaire for the household survey was conducted using tablet computers with the REDCap mobile application (Vanderbilt University, Nashville, USA). Data were stored in a secure REDCap server hosted by the University of Minnesota.

### Data analysis

The primary objective of this analysis was to determine the associations between housing factors on malaria infection. Participants with recent travel history were excluded from this analysis to exclude potential imported infections. Individual factors were considered in the analysis as potential confounders. These were individual age of the participant, sex of participant, education of the participant, and individual ITN use the previous night. The univariate associations between these individual factors and *P. falciparum* infection were determined using chi-squared tests for binary and categorical variables (sex, age category, education, ITN use) and Kruskal–Wallis tests for continuous variables (age).

Household structural variables that were investigated were the following: roof structure, wall structure, floor structure, windows, and eaves. Roofs, walls, and floors were collected as categorical variables based on the type of material as natural, rudimentary, or modern. For roofs natural materials were straw or leaves; rudimentary materials were bamboo, wood, or sticks; and modern materials were zinc, metal, or asbestos. For walls, natural materials were straw or mud; rudimentary materials were mud blocks, sticks, or metal; modern materials were cement, fired brick, or treated wood. For floors natural materials were soil or mud; rudimentary materials were bamboo, wood, or sticks; and modern materials were cement, brick, or tile. For analyses these were recategorized as rudimentary and modern, with natural and rudimentary materials being aggregated into a single category. Windows were collected as a categorical variable of whether they were open or able to close partially or fully. This variable was analyzed as whether windows were open or able to close or not (either partially or fully). Eaves were categorized as whether they were open or closed on the house. The aggregations were done to have results comparable to other studies and to improve sample sizes (2, 20).

Other household level variables were the number of residents per household or related to just the head of household. These were the highest level of education achieved by the head of household and whether the head of household primarily worked full-time, part-time, or seasonally. Occupation status was collected in this manner as many occupations in this area of the country are only part-time or seasonal work. These professions typically result in lower incomes compared to full-time work so this variable was used as a surrogate for household income level. The highest education level achieved by the head of household was categorized as primary or lower and secondary or higher for analysis. Occupation status of the head of household was categorized as full-time and part-time or seasonal for analysis.

Individual and household level characteristics of participants were compared by the household construction type (roof, walls, floor, windows, and eaves). Individual and household characteristics of participants were also compared by *P. falciparum* infection status by RDT.

Several generalized estimating equations (GEEs) logistic regression models were constructed to determine the independent effects of housing construction on malaria risk. GEE models were constructed using the household identification number as the cluster level variable to account for the non-independence of the exposure variables (housing construction) and spatial autocorrelation of the outcome variable (*P. falciparum* infection). Attention was made to potential confounding effects and the relationship between occupation and education and housing in the models. Unadjusted models were used to determine the associations between housing construction variables (roof type, wall type, floor type, windows, and eaves) and *P. falciparum* infection. Five unadjusted models were constructed using roof type, wall type, floor type, windows, and eaves respectively as the primary exposure with *P. falciparum* infection measured by RDT as the outcome. Fully adjusted GEE logistic regression models were used to determine the associations between housing construction variables while treating individual-level variables (age and ITN use the previous night) and household level variables (residents per household, head of household occupation time, and head of household education level) as confounders. These were also five separate models, one for each household construction variable as the primary exposure of interest (roof type, wall type, floor type, windows, and eaves) and *P. falciparum* infection as the outcome. Additional variables included in the model as confounders were age, ITN use the previous night, number of residents per household, head of household occupation time, and head of household education level. All potential confounders were decided *a priori* rather than using statistical significance in their individual association with the primary exposures or primary outcome.

Another set of GEE logistic regression models were constructed to investigate the relationship between head of household occupation and head of household education and household construction on malaria risk. These five models used the same exposures and outcome as above but were constructed by adjusting for confounding variables (age, ITN use the previous night, and residents per household) while leaving out head of household occupation and education hypothesizing that they were distal on the causal pathway and household construction mediated this relationship. Additionally, 10 separate GEE logistic mediation models were constructed to determine the proportion of effects mediated by household construction material in the relationship between head of household occupation and education and malaria risk (20, 23). In these models, head of household occupation and education were treated as the primary exposure variables and malaria infection was the outcome variable. The same confounding variables (age, ITN use the previous night, and residents per household) were included in all models. Household construction material was included as a mediator in each model. These were roof type, wall type, floor type, windows, and eaves. All analyses were conducted using R Studio (Version 2022.02.01).

## Results

### Demographics

**Figure 1:**
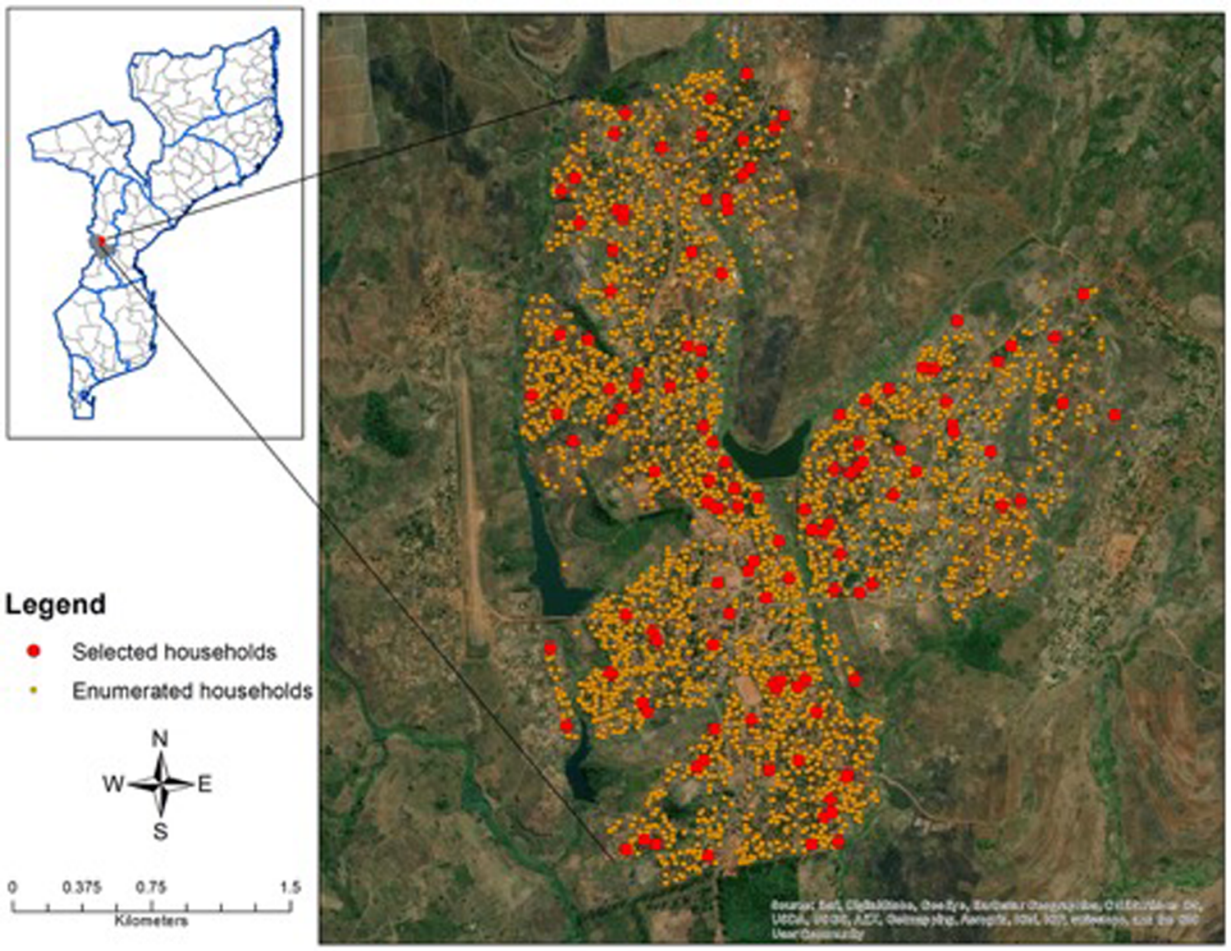
Map of the study area with enumerated and selected households.

**Figure 2:**
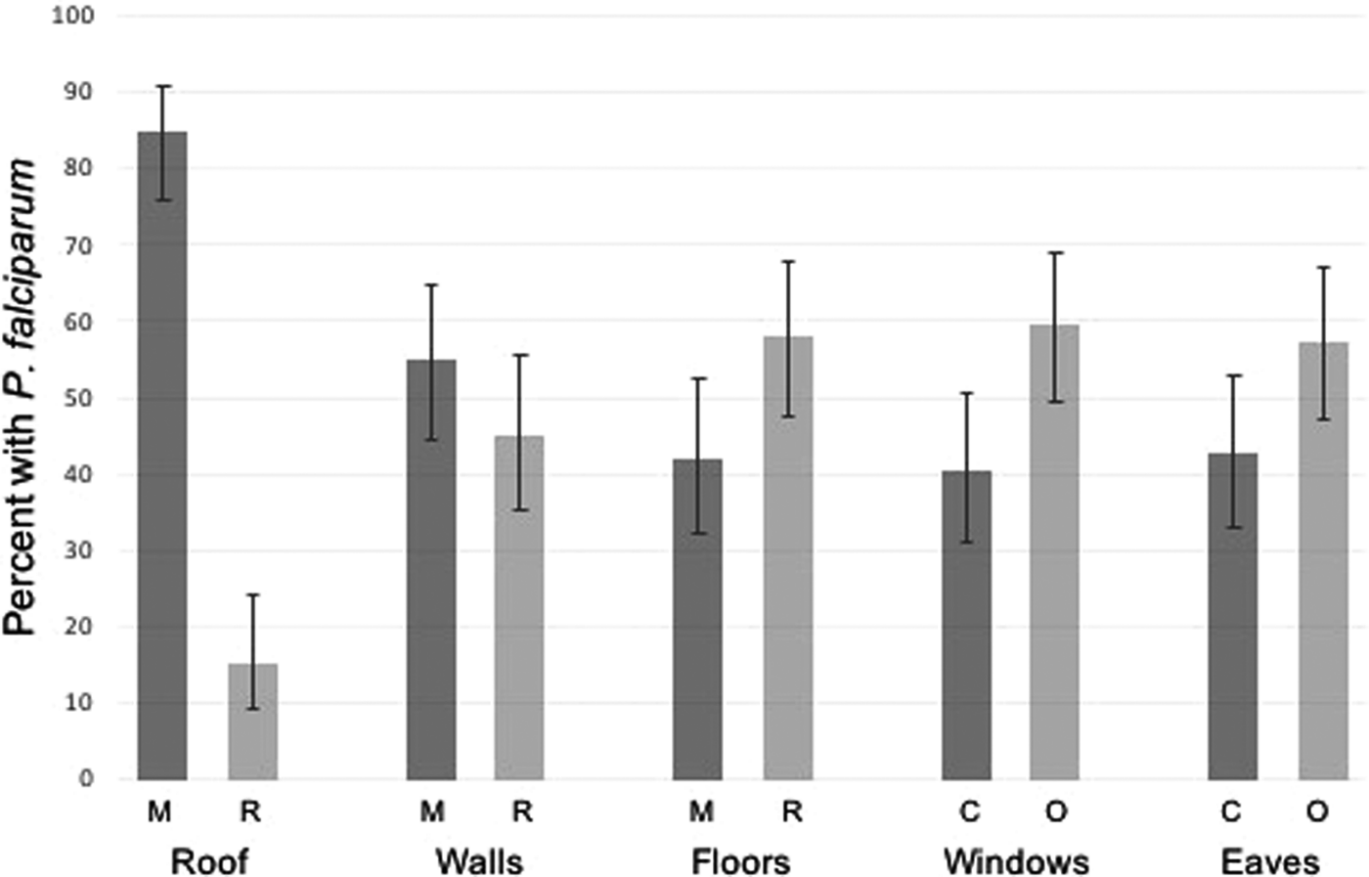
*P. falciparum* prevalence by housing structure. M, modern materials; R, rudimentary materials; C, closed/closable; O, open.

One-hundred and nine (109) households were approached to participate, with 83 household agreeing and completing the survey (Figure 1). The final analytic sample size consisted of 303 participants from 83 households. The overall malaria prevalence among the study population by RDT was 30.8%. When considering distributions of demographics between those positive and negative by RDT, continuous and categorical age was statistically significantly associated with *P. falciparum* infection (Table 1). Those in the 5- to 15-year-old age group had the highest malaria prevalence overall (Table 1). Occupation status of the head of household was also statistically significantly associated with *P. falciparum* infection with those having full-time employment having a lower risk of infection (Table 1). ITN use the previous night was found to be protective against *P. falciparum* infection (Table 1). Sex of the participant was not statistically significantly associated with *P. falciparum* infection (Table 1). Rudimentary floor structure and open eaves were associated with increased risk of *P. falciparum* infection (Figure 2). Roof and wall structure and windows were not statistically significantly associated with *P. falciparum* infection (Table 1 and Figure 2). Higher head of household education level was associated with *P. falciparum* infection while occupation status and number of residents per household were not associated with infection (Table 1).

**Table 1:** Characteristics of the study population.

	RDT positive (93)	RDT negative (209)
Age, median (IQR)	12 (7–19)	20 (11–31)
Age category, % (95% CI)		
<5	10.75 (5.84–18.98)	6.7 (3.99–11.02)
5–15	49.46 (39.33–59.64)	29.19 (23.39–35.75)
16–25	21.51 (14.23–31.14)	30.62 (24.72–37.24)
26–39	7.53 (3.59–15.09)	21.05 (16.03–27.15)
>39	10.75 (5.84–18.98)	12.44 (8.59–17.68)
Female, % (95% CI)	52.75 (42.38–62.88)	56.1 (49.19–62.78)
Education, % (95% CI)		
Primary	52.75 (42.38–62.88)	39.71 (33.27–46.54)
Secondary	20.88 (13.65–30.58)	24.88 (19.46–31.23)
Tertiary	26.37 (18.25–36.49)	35.41 (29.19–42.16)
Work, % (95% CI)		
Full time	48.39 (38.30–58.60)	69.89 (63.26–75.72)
Part time	36.56 (27.31–46.92)	21.53 (16.45–27.66)
Sometimes	15.05 (9.06–23.97)	8.61 (5.48–13.29)
ITN use, % (95% CI)	55.91 (45.58–65.76)	68.27 (61.60–74.27)
Residents, median (IQR)	6 (4–7)	4 (3–7)
Roof, % (95% CI)		
Modern	84.95 (76.03–90.94)	91.87 (87.27–94.90)
Rudimentary	15.05 (9.06–23.97)	8.13 (5.10–12.73)
Walls, % (95% CI)		
Modern	54.84 (44.53–64.75)	65.53 (58.75–71.74)
Rudimentary	45.16 (35.25–55.47)	34.47 (28.26–41.25)
Floor, % (95% CI)		
Modern	41.94 (32.24–52.30)	60.29 (53.46–66.73)
Rudimentary	58.06 (47.70–67.76)	39.71 (33.27–46.54)
Windows, % (95% CI)		
Open	40.42 (30.89–50.74)	25.84 (20.33–32.24)
Closeable	59.57 (49.26–69.11)	74.16 (67.76–79.67)
Eaves, % (95% CI)		
Open	42.55 (32.86–52.85)	33.65 (27.53–40.38)
Closed	57.45 (47.15–67.14)	66.35 (59.62–72.47)

When investigating the distributions of demographics by household structure head of household education was statistically significantly associated with roof, wall, and floor structure, as well as windows opening and eaves (Table 2). Head of household occupation status was statistically significantly associated with all household structural factors except for roof structure (Table 2). The number of residents in a household was statistically significantly associated with roof and floor structures, with modern roofs associated with more residents and modern floor structure associated with fewer (Table 2). Age, sex, and ITN use the previous night were not associated with household structural variables (Table 2).

**Table 2:** Participant characteristics by household structure.

	Roof structure		Wall structure		Floor structure		Windows		Eaves	
	Modern (278)	Rudimentary (31)	Modern (191)	Rudimentary (115)	Modern (172)	Rudimentary (137)	Open (92)	Closed (211)	Opened eaves (110)	Closed eaves (192)
Age, median (IQR)	17 (9–27)	20 (13–30)	17 (8–26)	19 (10–30)	18 (9–31)	17 (9–25)	17 (9–25)	18 (9–30)	18 (9–25)	17 (9–27)
Age category, % (95% CI)										
<5	8.27 (5.55–12.16)	6.45 (1.53–23.49)	8.38 (5.18–13.27)	7.83 (4.09–14.45)	7.56 (4.42–12.62)	8.76 (5.02–14.86)	9.78 (5.12–17.88)	7.11 (4.32–11.49)	8.18 (4.28–15.08)	7.81 (4.75–12.59)
5–15	36.69 (31.21–42.54)	19.35 (8.66–37.78)	37.7 (31.07–44.82)	30.43 (22.65–39.53)	35.47 (28.63–42.95)	34.31 (26.79–42.71)	34.78 (25.67–45.17)	36.02 (29.79–42.76)	33.64 (25.36–43.06)	36.98 (30.41–44.07)
16–25	27.34 (22.40–32.91)	38.71 (22.92–57.29)	26.18 (20.40–32.91)	32.17 (24.21–41.33)	25 (19.06–32.06)	32.85 (25.45–41.21)	32.61 (23.73–42.94)	25.59 (20.13–31.95)	33.64 (25.36–43.06)	24.48 (18.88–31.10)
26–39	16.19 (12.29–21.02)	19.35 (8.67–37.78)	17.8 (12.98–23.93)	13.91 (8.66–21.61)	20.93 (15.46–27.70)	10.95 (6.68–17.44)	10.87 (5.90–19.17)	19.43 (14.61–25.37)	13.64 (8.34–21.50)	18.23 (13.36–24.38)
>39	11.51 (8.24–15.85)	16.13 (6.62–34.27)	9.95 (6.42–15.11)	15.65 (10.04–23.58)	11.05 (7.13–16.72)	13.14 (8.40–19.96)	11.96 (6.69–20.45)	11.85 (8.11–16.98)	10.91 (6.26–18.33)	12.50 (8.50–18.01)
Female, % (95% CI)	55.72 (49.73–61.55)	45.16 (28.27–63.25)	54.3 (47.06–61.37)	53.98 (44.66–63.03)	54.76 (47.13–62.18)	54.48 (45.92–62.78)	58.24 (47.76–68.03)	53.88 (47.01–60.62)	52.78 (43.27–62.09)	56.38 (49.16–63.34)
Education										
Primary	41.67 (35.97–47.6)	67.74 (48.93–82.15)	34.39 (27.93–41.49)	61.74 (52.46–70.24)	25.88 (19.82–33.04)	67.15 (58.79–74.55)	60.87 (50.44–70.39)	36.36 (30.09–43.14)	50.91 (41.54–60.21)	39.47 (32.73–46.64)
Secondary	22.1 (17.58–27.40)	32.26 (17.85–51.07)	20.11 (14.96–26.47)	28.7 (21.11–37.71)	25.29 (19.29–32.42)	20.44 (14.45–28.09)	10.87 (5.90–19.17)	29.19 (23.39–35.75)	25.45 (18.12–34.51)	22.63 (17.20–29.17)
Tertiary	36.23 (30.75–42.10)	0	45.5 (38.50–52.69)	9.57 (5.34–16.54)	48.82 (41.34–56.36)	12.41 (7.82–19.13)	28.26 (19.92–38.42)	34.45 (28.29–41.18)	23.64 (16.55–32.57)	37.89 (31.24–45.04)
Work, % (95% CI)										
Full time	62.95 (57.09–68.45)	58.06 (39.70–74.44)	71.2 (37.94–56.17)	46.96 (37.94–56.17)	70.93 (63.66–77.27)	51.82 (43.41–60.14)	47.83 (37.72–58.12)	70.14 (63.59–75.96)	58.18 (48.68–67.11)	66.67 (59.66–73.01)
Part time	24.46 (19.75–29.88)	35.48 (20.35–54.21)	17.28 (12.53–23.35)	40 (31.38–49.29)	17.44 (12.44–23.90)	35.77 (28.13–44.20)	33.70 (24.69–44.06)	22.75 (17.56–28.93)	34.55 (26.18–43.99)	20.93 (15.64–27.20)
Sometimes	12.59 (9.17–17.05)	6.45 (1.53–23.49)	11.52 (7.69–16.91)	13.04 (7.98–20.61)	11.63 (7.60–17.38)	12.41 (7.82–19.13)	18.48 (11.73–27.87)	7.11 (4.32–11.49)	7.27 (3.65–13.97)	12.50 (8.50–18.01)
ITN use, % (95% CI)	66.06 (60.26–71.42)	54.84 (36.75–71.73)	66.32 (59.26–72.71)	61.74 (52.46–70.24)	69.59 (62.24–76.06)	59.12 (50.64–67.10)	70.65 (60.44–79.14)	61.43 (54.63–67.81)	62.73 (53.24–71.33)	64.93 (57.85–71.40)
Residents, median (IQR)	5 (3–7)	4 (2–6)	5 (3–7)	5 (3–7)	5 (3–7)	6 (4–8)	4 (3–7)	5 (3–7)	5 (3–8)	5 (3–7)
RDT results, % (95% CI)										
Positive	29.15 (65.13–75.97)	45.16 (28.27–63.25)	27.42 (21.45–34.32)	37.17 (28.69–53.48)	23.64 (17.73–30.77)	39.42 (31.53–47.90)	41.30 (31.60–51.73)	26.54 (20.99–32.94)	36.36 (27.84–45.84)	28.13 (22.19–34.94)
Negative	70.82 (24.03–34.87)	54.84 (36.75–71.73)	72.58 (65.68–78.55)	62.83 (53.48–71.31)	76.36 (69.23–82.27)	60.58 (52.10–68.47)	58.70 (48.27–68.40)	73.46 (67.06–79.01)	63.64 (54.16–72.16)	71.88 (65.06–77.81)

### Statistical models

The results of the GEE models accounted for the spatial correlation in both sampling from the same household and having household level variables. In the completely unadjusted models roof structure, wall structure, floor structure, and windows were statistically significantly associated with increased risk in *P. falciparum* infection. Rudimentary roofs were associated with a 1.98 (CI: 0.93–4.20) times increased odds of malaria infection compared to modern roofs. Rudimentary walls were associated with a 1.6 (CI: 0.98–2.64) times increased odds in malaria infection compared to modern walls. Rudimentary floors were associated with a 2.14 (CI: 1.30–3.51) times increased odds in malaria infection compared with modern floors. Completely open windows were associated with a 1.96 (1.16–3.26) times increased odds in malaria infection compared with closable windows (Table 3). In the model adjusting for all individual (age, ITN use the previous night) and household (residents per household, head of household occupation, and head of household education) factors as potential confounders, roof structure was the only household structural variable that was statistically significantly associated with increased malaria risk. Rudimentary roof structure was associated with a 2.41 (CI: 1.03–5.63) times increased odds of *P. falciparum* infection compared to modern roof structure. The effect size of the estimates for wall structure, floor structure, windows opening, and eaves were similar in the fully adjusted model, but with decreased statistical significance.

**Table 3:** Unadjusted and adjusted models of the association between household construction and *P. falciparum* infection. Adjusted models are adjusted for confounders and potential mediators (occupation and education).

	Unadjusted			Adjusted		
	OR	CI	*p* value	OR	CI	*p* value
Roof type						
Modern	REF					
Rudimentary	1.98	0.93–4.20	0.08	2.41	1.03–5.63	0.04
Wall type						
Modern	REF					
Rudimentary	1.6	0.98–2.64	0.06	1.43	.79–2.57	0.236
Floor type						
Modern	REF					
Rudimentary	2.14	1.30–3.51	0.003	1.61		0.126
Windows						
Closed	REF					
Open	1.95	1.16–3.26	0.011	1.69	0.92–3.12	0.091
Eaves						
Closed	REF					
Open	1.46	0.89–2.41	0.138	1.43	0.82–2.47	0.204

In separate models adjusting for all confounders (age, ITN use the previous night, and residents per household) leaving out head of household occupation and head of household education all household structural variables were statistically significantly associated with increased risk of malaria infection with the exception of open eaves. In these models, rudimentary roofs were associated with a 2.46 (CI: 1.10–5.49) times increased odds in malaria infection, rudimentary walls were associated with a 1.75 (CI: 1.03–1.03–2.97) times increased odds in malaria infection; rudimentary floors were associated with a 1.94 (CI: 1.15–3.28) times increased odds in malaria infection; and completely open windows were associated with a 1.95 (CI: 1.16–3.49) times increased odds in malaria infection. All these effect sizes were similar to those in the unadjusted analysis except roof type, which was similar to the effect size in the fully adjusted model that included occupation and education.

The results for the GEE mediation model accounted for the spatial correlation to determine the proportion of the relationships between head of household occupation and education and *P. falciparum* infection that is mediated by household structure (Table 4). In these models we found no statistically significant evidence of mediation by household structure in these relationships (Table 4). The percentages mediated by household structure were higher for all household structural variables for head of household education level compared to head of household occupation level. This aligns with head of household education being associated with *P. falciparum* infection and being associated with all household structural variables.

**Table 4:** Unadjusted and adjusted models of the association between household construction and *P. falciparum* infection. Adjusted models are adjusted for confounders only without potential mediators (occupation and education).

	Unadjusted			Adjusted			Percent mediated—occupation	Percent mediated—education
	OR	CI	*p* value	OR	CI	*p* value		
Roof type							2.32 (−81.6–86.2)	17.6 (−112.3–143.0)
Modern	REF							
Rudimentary	1.98	0.93–4.20	0.08	2.46	1.10–5.49	0.03		
Wall type							10.8 (−70.1–92.4)	28.1 (−96.4–153.2)
Modern	REF							
Rudimentary	1.6	0.98–2.64	0.06	1.75	1.03–2.97	0.04		
Floor type							12.5 (−68.1–92.3)	43.9 (−96.1–152.4)
Modern	REF							
Rudimentary	2.14	1.30–3.51	0.003	1.94	1.15–3.28	0.013		
Windows							13.4 (−68.8–94.6)	16.4 (−108.7–140.6)
Closed	REF							
Open	1.95	1.16–3.26	0.011	2.01	1.16–3.49	0.013		
Eaves							3.52 (−80.4–87.1)	8.96 (−121.2–138.3)
Closed	REF							
Open	1.46	0.89–2.41	0.138	1.5	0.89–2.53	0.129		

## Discussion

Our study found convincing evidence of housing structure being associated with malaria risk. After accounting for individual-level confounding factors, these associations held. We also found some evidence that this may be a mediating association between head of household work status and education level; however, these associations were not statistically significant. When these factors were included in the models, rudimentary roof structure was statistically significantly associated with increased risk of *P. falciparum* infection. It is likely that the associations between head of household work status and education level and malaria risk are partially mediated by household structure.

Our findings are consistent with results from other areas in sub-Saharan Africa. Sikalima et al. found evidence that housing structure mediates the relationship between income and malaria risk in northern Zambia (20). While we did not find statistically significant evidence of mediation, it is likely that there is partial mediation. Our study also did not have income, but used full-time vs. part-time work as a surrogate for household income. Head of household education level also acts as a proxy for potential earning, and acts independently as potential health and scientific knowledge base.

The most consistent finding in our study was that roof structure was statistically significantly associated with risk of *P. falciparum* infection (2, 20, 24, 25). Rudimentary roof structures were consistently associated with increased odds of infection. This finding has been observed across multiple studies (2, 13, 16, 20, 26). An interesting finding was also the consistency of floor structure being associated with malaria risk. This is a finding that has been shown across other settings but is not always consistently associated with malaria risk (2, 16). This finding is interesting in this setting as households are not built on stilts and having rudimentary flooring is not thought to provide additional access to mosquito entry into the house. However, having rudimentary flooring could change the microclimate of the house which may be more suitable for the vectors (27, 28). Our finding that having non-closable windows being associated with increased risk of malaria infection is expected as open windows provide access for mosquito entry into the house. While having rudimentary roofs can provide a suitable environment for mosquitos to rest they also can alter the indoor climate of the household. These specific structure types and their associations with *P. falciparum* infection provide insights into the potential transmission dynamics and mosquito environmental preferences.

As this was an observational, cross-sectional study the results cannot be interpreted as causal. However, these results have been consistent across different settings and should be further investigated as the primary focus of the research questions, rather than as part of pilot studies looking into malaria risk factors in communities. Additionally, specific factors related to housing structure, such as holes in ITNs, the color of housing materials, and time spent indoors and outdoors during mosquito feeding times were not included in these models. These may be important factors and should be considered in future studies. Future research should focus specifically on different housing structure types with larger sample sizes and include a longitudinal aspect to make more firm conclusions.

However, with such consistent results, household improvements should be considered as a potential malaria prevention intervention. While some interventions focused on improving household structure have been studied in cluster randomized trials, these have often been small with inconclusive results (14, 18, 29). Larger trials across multiple sites should be explored to best inform a package of interventions that will have a more long-lasting impact on reducing malaria transmission. Improved housing structure is one of the few interventions that provides a lasting impact, particularly in areas like rural Mozambique that only conduct IRS and/or ITN distributions at irregular intervals and need to continue to be done to maintain effectiveness.

In 2020 Manica Province conducted a mass ITN distribution of Interceptor® nets. This distribution was associated with a decline in malaria incidence, however cases have since rebounded. This is not uncommon in high transmission settings. The need for expanding malaria prevention tools is highlighted by these types of events. Many interventions provide short-lasting results, but without continued distribution and investment sustainable declines in transmission have been fleeting. Improvements in housing may be an area of malaria prevention that can have more long-lasting and sustainable impacts on local transmission.

Additionally, this area of Mozambique experiences frequent severe weather events, including tropical storms and cyclones (30). These events typically result in household structural damage. Acknowledging the associations between household structure and malaria risk will be important in responding to severe weather events, with a particular focus on repairing this damage and improving housing structure overall.

## Data Availability

The raw data supporting the conclusions of this article will be made available by the authors, without undue reservation.
